# A Cost-Effectiveness Analysis of Pre-Exposure Prophylaxis to Avert Rabies Deaths in School-Aged Children in India

**DOI:** 10.3390/vaccines11010088

**Published:** 2022-12-30

**Authors:** Abhishek Royal, Denny John, Omesh Bharti, Ritesh Tanwar, Deepak Kumar Bhagat, Retna Siwi Padmawati, Vishal Chaudhary, Reddicherla Umapathi, Pradeep Bhadola, Adi Utarini

**Affiliations:** 1 Faculty of Medicine, Public Health and Nursing, Universitas Gadjah Mada, Yogyakarta 55281, Indonesia; 2 Faculty of Life and Allied Health Sciences, Ramaiah University of Applied Sciences, Bengaluru 560054, India; 3 State Institute of Health and Family Welfare, Department of Health & Family Welfare, Government of Himachal Pradesh, Shimla 171009, India; 4 Directorate of Health Services, Government of Madhya Pradesh, Bhopal 462002, India; 5 Centre of Social Medicine and Community Health, Jawaharlal Nehru University, Delhi 110067, India; 6 Department of Physics, Bhagini Nivedita College, University of Delhi, Delhi 110021, India; 7 Department of Biological Sciences and Bioengineering, Inha University, Incheon 22212, Republic of Korea; 8 Centre for Theoretical Physics and Natural Philosophy, Mahidol University, Nakhonsawan Campus, Phayuha Khiri, NakhonSawan 60130, Thailand

**Keywords:** cost effectiveness, India, rabies, school-aged children, public health, child health

## Abstract

Children contribute to one-half of the total painful rabies mortalities in India. The state-of-the-art rabies mortality averting strategies need exploration for the effective implementation of pre-exposure prophylaxis (PrEP) in India. This study reports on the economic evaluation of various PrEP and post-exposure prophylaxis (PEP) strategies to avert rabies mortalities in school-aged children in India. A decision tree model has been developed for children in the age group of 5–15 years to evaluate various PrEP + PEP and PEP only regimens. The 2-site intradermal regimen administered on day zero and seven was chosen as the intervention [PrEP (I)]. ICER was calculated from the quasi-societal and quasi-health systems’ perspectives for the base case analysis, along with one-way sensitivity, and scenario analyses for each regimen. The incremental DALYs averted per million population with the implementation of PrEP (I) ranged between 451 and 85,069 in 2020. The ICER was reported in the range of USD 384–352/DALY averted (non-dominant) in comparison to PEP regimens from a quasi-societal perspective. PrEP (I) is reported to be ‘very cost effective’ in comparison with PEP regimens from the quasi-societal and quasi-health systems’ perspectives and reduce deaths by up to 89.9%. This study concludes that the PrEP (I) regimen is a cost-effective and life-saving strategy to avert painful mortalities due to rabies in school-aged children in India.

## 1. Introduction

Rabies is a zoonotic disease caused by a lyssavirus infection. The causative agent is an RNA virus, and the infection leads to fatal encephalitis. The earliest effective vaccine against rabies was developed by Louis Pasteur in 1885 and the current vaccines are highly effective when given pre- or post-exposure to a bite from a potentially rabid animal and are accompanied by minimal local side-effects. Therefore, the disease can be prevented through the well-timed administration of post-exposure prophylaxis (PEP) following animal bites [1,2]. The effective strategy to prevent rabies also includes the administration of pre-exposure prophylaxis (PrEP) in high-risk individuals, including veterinary healthcare workers, forest dwellers, animal handlers, children, and adults at risk [1].

It is an endemic and major public health problem in India. Annual human deaths due to rabies are estimated to be around 20,000 and the annual incidence of dog bites is estimated to be 1.7% (17.5 million per year) in India [3]. Hence, India contributes to approximately one-third of the global rabies burden annually. The disease mainly affects people belonging to a low socio-economic status and children in the age group of 5–15 years in the country [4]. A systematic review on dog-mediated rabies in India reported that the majority of the dog bites (>60%) in children below 15 years of age are experienced by children in the age group of 7–12 years. Moreover, children (<15 years of age) are more vulnerable (58%) to experiencing 2–4 wounds per encounter. This could be related to their shorter stature and lesser strength to scare away the dogs and may lead to extensive bites, requiring operative interventions and thereby resulting in a greater morbidity and pain associated with bites and their treatment [4].

The high costs associated with the administration of rabies immunoglobulins (RIG), extended doses of vaccination for PEP, and limited availability of RIG have been identified as crucial reasons for the low coverage of complete PEP, ultimately resulting in a significant number of deaths [5]. Moreover, RIG infiltration into wounds is highly painful, especially if wounds are on sensitive parts, such as the face and hands as is commonly seen in children.

PrEP has great potential to save thousands of children from painful rabies deaths and reduce DALYs (disability adjusted life years) in India. The study is a cost-effectiveness analysis to understand the costs, cost effectiveness, and utilization of various PrEP + PEP and PEP only strategies to avert rabies deaths in school-aged children in India.

## 2. Materials and Methods

### 2.1. Model Structure

**Figure 1:**
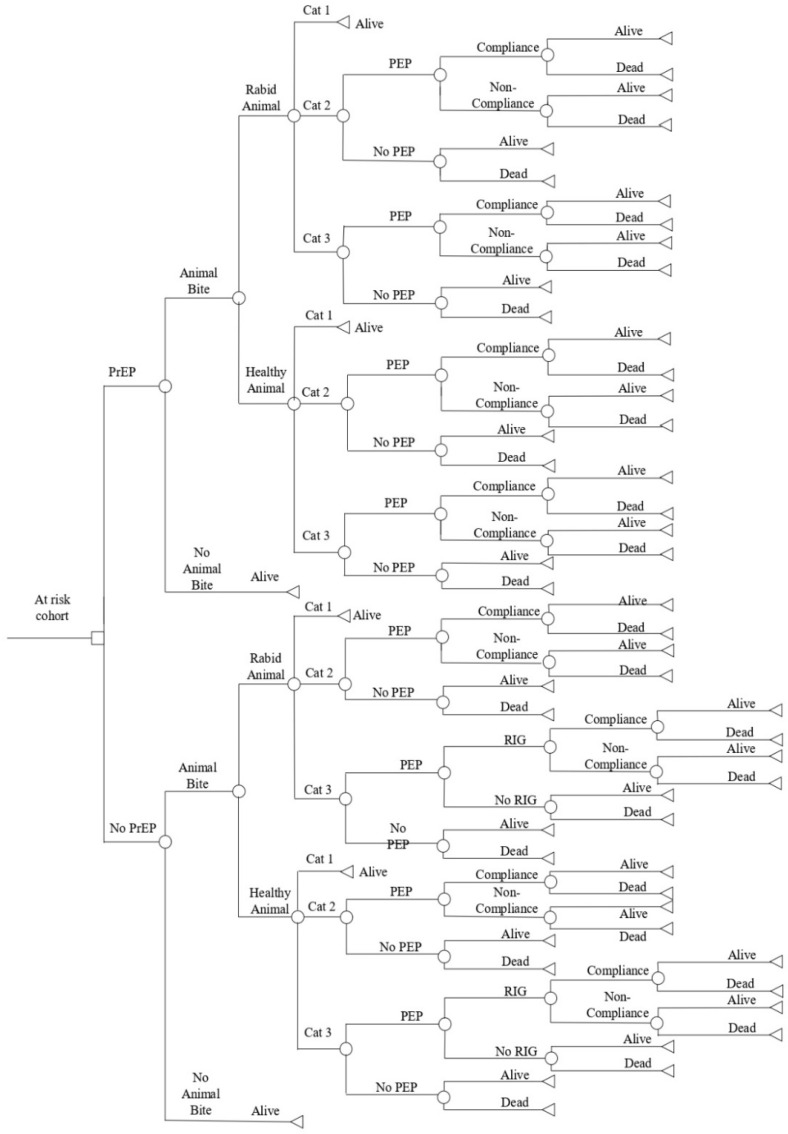
Decision Tree Model. Cat 1—Category 1, Cat 2—Category 2, Cat 3—Category 3, PEP—Post-Exposure Prophylaxis, PrEP—Pre-Exposure Prophylaxis, RIG—Rabies Immunoglobin.

A static decision tree model developed to estimate the global burden of canine rabies was adapted for children in the age group of 5–15 years (school-aged children) in the Indian setting and built in the plant-a-tree MS Excel add-in in MS Excel 2013 [6,7] (Figure 1). The decision tree compared the costs involved in the implementation of various PrEP + PEP and PEP only strategies with rabies-associated deaths and DALYs (disability-adjusted life years) in India in the study cohort. The analysis was conducted from the quasi-societal and quasi-health systems’ perspectives in the time horizon of 2020.

The WHO-GDP based CET (cost-effectiveness thresholds) approach suggested by the Commission for Macroeconomics on Health (2001) was used to report the cost-effectiveness interventions with an incremental cost per DALY averted less than the per capita GDP (in low middle-income countries (LMICs) are “very cost effective”, and those costing less than triple the per capita GDP are “cost- effective”) [8]. The per capita GDP for the CET threshold for the financial year 2020 for India is INR 145,679 or USD 1965 [9].

The study has been conducted and reported in adherence to the Consolidated Health Economics Evaluation Reporting Standards (CHEERS) checklist for full economic evaluations) [10].

### 2.2. Model Data Inputs

The model was populated with the data inputs that were extracted from an independent review of published and grey literature, national representative surveys, programmatic reports, and national and state level databases. The experts’ opinions and logical assumptions were considered for the data inputs that were not available in the literature. The literature reporting the relevant data points from the most recent, community-based studies conducted on the target population were chosen for this research study. The data inputs and assumptions were validated by the field experts.

### 2.3. Vaccine Schedule

As compliance is higher in shorter regimens, a 2-site intradermal (ID) PrEP schedule administered on day zero and seven and a four-site ID regimen on day zero as PEP in the healthcare facility was chosen to be the intervention (PrEP I) in this study [11]. All other regimens of PrEP + PEP and PEP alone, as recommended in the WHO and national guidelines, were chosen as comparators for the cost-effectiveness analysis [11,12] (Table 1).

### 2.4. RIG Schedule

The RIG is administered in category III exposures in previously unvaccinated individuals at the first visit. The administration of RIG in category III exposures was considered in all the PEP only regimens, as no previous exposure to vaccination was assumed. The administration of equine rabies immunoglobulins (ERIG) was considered for the base-case analysis, as it is easily available, less costly, and equally effective as human rabies immunoglobulins (HRIG) and rabies monoclonal antibodies (R-Mab). Moreover, the local plus systemic administration of ERIG was considered, as it is the most prevalent practice in India [5].

### 2.5. Epidemiological Data

#### 2.5.1. Population Size

A hypothetical cohort of 1-million children was populated in each branch at the decision node of the model for calculations.

#### 2.5.2. Annual Bite Incidence

A community-based cross-sectional study reported a dog-bite incidence of 44 in 1000 children in the age group of 5–14 years [13]. The time frame of the study was one year and, therefore, only one animal bite per year was considered for all the bite victims.

#### 2.5.3. Rabies Positivity

The health facility survey component of the WHO-APCRI Survey 2017 reported that 29.5% of the biting animals in all the bite victims presented to the health facility showed some signs of suspected rabies [5]. Therefore, the probability of rabies positivity in the biting animal was assumed to be 0.295 for the base-case analysis.

#### 2.5.4. Distribution of Category of Exposure

A cross-sectional study conducted in the anti-rabies vaccination (ARV) clinic in a tertiary care centre in the Solapur district reported the profile of animal bite cases in children in2016 [14]. The category of exposure was reported to be 0.53%, 26.71%, and 72.76% in category I, II, and III exposures, respectively, in the age group of 5–15 years.

#### 2.5.5. Risk Probability of Rabies after Exposure

The probability of the development of rabies after a category I exposure from a rabid animal was assumed to be zero. The administration of an adequate amount of an anti-rabies vaccination through any route after category II or category III exposures and full compliance to the ARV regimen along with timely and adequate administration of RIG in category III exposures in previously unvaccinated children was considered to be 100% effective, and the probability of death due to rabies is assumed to be zero, even after exposure to a rabid animal.

The probability of developing rabies in the category II/III exposure in the absence of the administration or in the absence of the full administration of the PEP vaccination was considered to be0.19, as reported in a study from Tanzania [15]. However, the probability of developing rabies in the category III exposure in the absence of the administration of RIG in previously unimmunized individuals was assumed to be 0.30, as per the expert’s opinion.

#### 2.5.6. Practice of Seeking PEP after Animal Bite

The practice of seeking PEP has been reported to be 88.9% in community surveys in the WHO-APCRI survey 2017 [5]. This was assumed to be the PEP seeking behaviour in the base-case analysis.

#### 2.5.7. RIG Administration

The ERIG was reported to be administered in the 13.6% category III exposures in community surveys in the WHO-APCRI Survey 2017 [5]. This estimate was used, as the administration of ERIG was considered for the base-case analysis.

#### 2.5.8. PEP Compliance

The compliance rate for a complete course of PEP through the intra-muscular (IM) route (five doses or visits) and intra-dermal (ID) route (four doses or visits) was reported to be 65.9% and 85.1%, respectively, in health facility surveys conducted by WHO-APCRI [5]. The survey also reported the compliance for every visit for both routes of PEP administration. The compliance per visit was used as a proxy indicator to assume compliance for the various regimens, according to the number of visits.

#### 2.5.9. Duration of Protection

The duration of protection was considered to be 20 years, as reported in a study [16].

#### 2.5.10. Average Time for Death after Onset of Rabies

The average time for death after the onset of the symptoms of rabies was assumed to be 5 days in this study.

### 2.6. Health Resource Utilization and Cost Inputs

The dose of the vaccine utilized during vaccination is taken according to the route of administration and is the same for both pre-exposure as well as post-exposure prophylaxes. The maximum dose of ERIG administered is 40 IU/kg weight, according to the guidelines [11]. As children in the age group of 5–15 years were the target population in this study, the average age-specific proportionate weights were calculated from the data reported by the Census of India (2011), the National Family Health Survey (NFHS-4) 2015–16, and the Indian Growth References were used for the calculation of the average dose of RIG in the study [17,18,19].

The procurement cost of one vaccine vial for public facility in the state of Madhya Pradesh (MP), as reported in the rate contract (2020), and the average procurement cost for a 5 mL vial of ERIG in the study states, as reported in WHO-APCRI survey, were used in the study [5,20].

The median hospital charges per visit, as reported in the WHO-APCRI survey, and the procurement costs of syringes (per unit) and gloves (per pair) in 2020, as reported from the state of Himachal Pradesh, were used in the current study [5]. The costs of other medicines and consumables for the 1st PEP visit were calculated from the costs reported in the WHO-APCRI Survey [5]. The costs of human resources per visit were calculated from the data received from the ARV clinic, Shimla and National Health System Cost Database of India [21].

As the palliative care of a patient after the onset of rabies requires intensive care in an isolated dark room, the cost for ‘Intensive Care services without ventilator’ as per the Pradhan Mantri Jan Arogya Yojana (PMJAY) Health benefit Package 2.0 was used as a proxy for the costs associated with the treatment of fatal rabies [22]. The per capita program management costs were calculated from a study on rabies control interventions conducted in the state of Tamil Nadu [23].

The travel and meal costs for the patient/client, along with one attendant, were extracted from the WHO-APCRI Survey [5]. As the target population is an economically non-productive population, only the loss of wages for one attendant was considered in the study and the average state-wise per day wage rate for unskilled manual workers under MGNREGA 2020 (Mahatma Gandhi National Rural Employment Guarantee Act 2005) was assumed for the loss of wages per attendant [24].

The wastage factor for the vaccine and RIG was considered to be 30% and 15%, respectively, as reported in a study in the state of Tamil Nadu [23].

All costs were converted into 2020 Indian national rupee and international dollars’ values using implicit price deflators for purchasing power parities, as recommended by Campbell & Cochrane Economic Methods Group (CCEMG) [25]. All of the costs and utilities were discounted at 3% following the WHO guide for standardization of economic evaluations of immunization programmes [26].

The calculations for the direct medical costs included the costs of the vaccine, RIG, consumables (syringes and gloves), other medicines, human resources, and hospital charges. It also included the costs associated with the wastage of vaccines and RIG. The calculations for direct non-medical costs included transportation, meals, and program management costs and the indirect cost only included the loss of wages in this study.

### 2.7. Utilities

Disability-adjusted life years (DALYs): the DALYs were calculated in the study using the following equation:

DALY = YLL + YLD

where:

- YLL stands for years of life lost due to premature mortality. It is calculated using the following formula:

YLL = Number of deaths × standard life expectancy at age of death

- YLD stands for years lived with disability. It is calculated using the following formula:

YLD = number of new cases of a disease × disability weight × the average time a person lives with the disease before death

For YLL calculations, the life expectancy at the age of 10 years was used as a proxy for calculations, as per the data reported in SRS based abridged life tables (2014–18) [27].

For YLD calculations, the number of rabies deaths is used as a proxy for number of new cases, as rabies is almost 100% fatal after its onset. The disability weight reported in a self-administered web-based survey in Korea was used for the calculation of YLD [28]. The DALYs were also discounted at 3%, according to the WHO guide for the standardization of economic evaluations of immunization [26]. The data inputs are tabulated in Table 2.

### 2.8. Sensitivity and Scenario Analyses

#### 2.8.1. One-Way Sensitivity Analyses

A one-way sensitivity analysis was conducted to test the uncertainty of the variables on the outcomes. The upper and lower limits of the data inputs extracted from the literature were taken for one-way sensitivity analyses in the study [5,15,24,30,31,32,33]. For variables where no additional data inputs were available in the literature, the upper and lower limits were set at varying degrees of variation, as per the feasibility of the values (see Appendix A). Tornado diagrams were plotted for representing this analysis.

#### 2.8.2. Scenario Analysis

Three different regimens of RIG were assessed for the scenario analysis for their cost-effectiveness. The updated WHO guidelines recommend a local infiltration of RIG into wounds with no systemic infiltration of the remaining RIG [1]. Therefore, the local infiltration of RIG into wounds only in category III exposures in previously unvaccinated children was assessed in the scenario analysis.

The local and systemic infiltration of human rabies immunoglobulins (HRIG) was also assessed in the scenario analysis. The average cost of procurement of HRIG was taken as INR 3700 per vial, and access to HRIG administration was considered in 4.5% category III exposures, as reported in the community survey in WHO-APCRI Survey 2017 [5].

The recent advancement proves the high potential of the local infiltration of rabies monoclonal antibodies (R-Mab) in category III exposures as a means of passive immunization in previously unvaccinated individuals due to its high potency, purity, and easy operationalization over RIG [5]. The local administration of R-Mab was also assessed in the scenario analyses. As R-Mab is currently under market surveillance, the access to R-Mab was considered to be 1% only. The market prices of R-Mab were used for the calculations [5].

## 3. Results

### 3.1. Base Case Analysis

#### 3.1.1. Cost-Effectiveness of PrEP (I) vs. Other PrEP Strategies

PrEP (I) is expected to prevent 122, 13, and 13 additional deaths per million population in one year in comparison to C1, C2, and C3 PrEP strategies. Around 4236, 451, and 451 more DALYs can be prevented per million population per year due to the implementation of PrEP (I) over C1, C2, and C3 strategies, respectively. The implementation of PrEP (I) was reported to be cost effective over the C1 PrEP strategy, while not reported to be cost-effective over other (C2 and C3) PrEP strategies from the quasi-societal perspective (Table 3).

From the quasi-health systems’ perspective, the implementation of PrEP (I) was reported to be very cost-effective over the C1 strategy. However, the implementation of PrEP (I) was not reported to be cost-effective over C2 and C3 PrEP strategies from the quasi-health systems’ perspective (Table 3). The ICER is dominant for the cost-effectiveness analysis of PrEP strategies.

#### 3.1.2. Cost-Effectiveness of PrEP (I) vs. PEP Strategies

PrEP (I) is expected to avert 2251, 2296, 2334, 2450, and 2450 additional deaths due to rabies per million population in one year, in comparison to C4, C5, C6, C7, and C8 PEP strategies. Around 78,159, 79,721, 81,041, 85,069, and 85,069 additional DALYs could be prevented per million population per year due to the implementation of PrEP (I) over C4, C5, C6, C7, and C8 strategies, respectively. Although the reported ICER was non-dominant, the implementation of PrEP (I) was reported to be very cost-effective over all PEP strategies from the quasi-societal perspective (Table 3).

The calculated values of ICER in the quasi-health systems’ perspective was further lesser than the quasi-societal perspective. The ICER was also non-dominant in the quasi-health systems’ perspective; the implementation of PrEP (I) was reported to be very cost-effective over all PEP strategies (Table 3).

### 3.2. Sensitivity Analyses

The cost-effectiveness of PrEP (I) strategy over other PrEP strategies in the cohort was sensitive to the following parameters: (1) PEP compliance; (2) rabies positivity of the biting animal; (3) risk probability of rabies in a category II/III exposure in the absence of the administration or in the absence of the full administration of the PEP vaccination; (4) the animal bite incidence; and (5) the loss of wages of the attendant; and (6) the cost of the vaccine vial.

The cost-effectiveness of the PrEP (I) strategy over PEP strategies in the cohort was sensitive to the following parameters: (1) rabies positivity of the biting animal; (2) the risk probability of rabies in category III exposures in previously unvaccinated children in the absence of RIG; (3) the animal bite incidence; (4) the category of exposure; (5) the loss of wages of attendant; and (6) PEP seeking behaviour.

From a quasi-societal perspective, the PrEP (I) strategy was found to be very cost-effective in all of the results of the sensitivity analysis involving PEP strategies (C4–C8). The PrEP (I) strategy was not found to be cost-effective in all of the results of the sensitivity analysis involving the C3 PrEP strategy. PrEP (I) was also not found to be cost-effective in all of the results of the sensitivity analysis involving the C2 PrEP strategy, except when the PEP compliance reduces to 94.4%. The PrEP (I) strategy was found to be cost-effective or very cost-effective in all of the results of the sensitivity analyses involving the C1 PrEP strategy, except when the rabies positivity of the biting animal reduces to 8.2%. Moreover, the PrEP (I) strategy was found to be very cost-effective in sensitivity analyses involving the C1 PrEP strategy when: (1) the probability of rabies positivity of the biting animal is as high as 1; (2) PEP compliance in the bite victims reduces to 89.4%; and (3) the risk probability of rabies in a category II/III exposure in the absence of the administration or in the absence of the full administration of PEP vaccination rises to 0.28.

**Figure 2:**
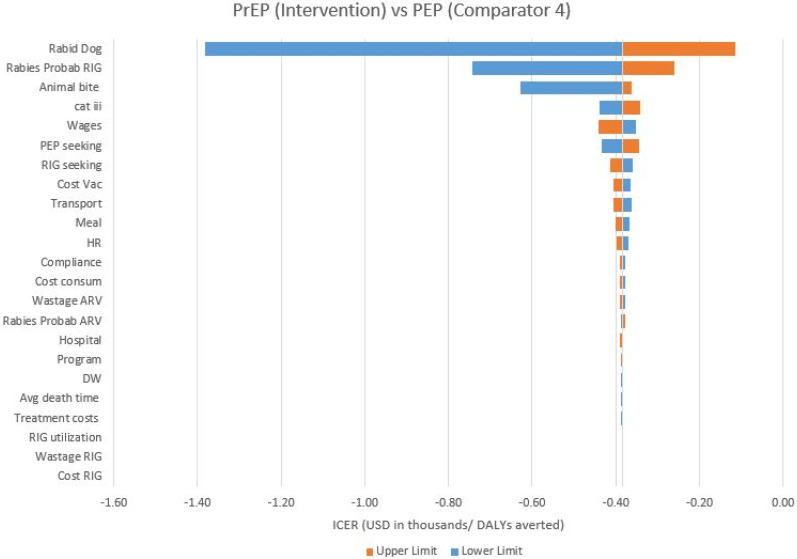
One−way sensitivity CE analysis: PrEP (I) vs. C4. Animal bite—Animal bite incidence; Avg death time—Average time a person lives after the onset of rabies; Cat iii—Category of exposure; Compliance—Compliance for full course of PEP; Cost consum—Cost of consumables; Cost RIG—Cost per RIG vial; Cost vac—Cost per vaccine vial; DW—Disability weight; Hospital—Hospital charges; HR—Costs associated with human resources; Meal—Meal cost for the patient/client and one attendant (per visit); PEP seeking—PEP seeking behaviour of the bite victims; Program—Program management costs; Rabid dog—Rabies positivity of the biting animal; Rabies Prob ARV—Probability of developing rabies in Cat II/III exposure in absence of administration or full administration of PEP vaccination; Rabies Prob RIG—Risk probability of rabies in category III exposures in previously unvaccinated children in absence of RIG; RIG seeking—RIG administration in Cat III exposures; RIG utilization—Utilization of calculated dose of RIG; Transport—Travel cost for the patient/client and one attendant (per visit); Treatment—Treatment cost of fatal symptomatic rabies; Wages—Loss of wages of attendant; Wastage ARV—ARV wastage; Wastage RIG—RIG wastage.

A tornado diagram of the one-way sensitivity analysis of PrEP (I) and C4 comparator is presented in Figure 2 (see Appendix A for all of the tornado diagrams).

### 3.3. Scenario Analyses

The scenario involving the local infiltration of ERIG into wounds only in category III exposures reported no change in the ICER with the comparators 4, 5, 6, 7, and 8 in both the quasi-societal and quasi-health systems’ perspective.

The scenario comprising the use of HRIG reported a greater cost-effectiveness of PrEP (I) over the comparator PEP regimens in both perspectives. The ICER was further lesser in IM comparators than ID comparators and PrEP (I) has been reported as being more cost-effective in the scenario of HRIG in PEP regimens involving the intra-muscular route of vaccination.

The third scenario involved the use of R-Mab in category III exposures and again reported a greater cost-effectiveness of PrEP (I) over the comparator PEP regimens in both perspectives. However, the PrEP (I) strategy has been proven to be even more cost-effective in the scenario of using R-Mab than the scenario involving the use of HRIG. The ICER was further lesser in IM comparators than ID comparators. The detailed results are tabulated in Table 4.

## 4. Discussion

A systematic review published in the WHO Bulletin in 2017 reported that PrEP could be beneficial in settings with delayed or no access to PEP and RIG and a high risk of exposure and chances of exposure to remain unnoticed (for example in young children) [29]. The bites in children commonly go unreported and unrecognized and, as a result, 30–60% of reported rabies deaths occur in children below 15 years of age in India [34,35]. Therefore, PrEP could be a beneficial strategy to avert rabies deaths in school-aged children in India.

The PrEP (I) strategy was reported to be very cost-effective from the quasi-societal and quasi-health systems’ perspectives over all IM and ID PEP only strategies in children in the age group of 5–15 years in India. The strategy has not been reported to be cost-effective over IM PrEP strategies from both perspectives in the cohort. Though higher incremental costs and non-dominant ICER has been reported with the PrEP (I) strategy over PEP strategies, the strategy has been proven to be very cost-effective due to high DALYs as a result of a significant number of premature deaths associated with PEP strategies in children. These deaths in the PEP strategies can be attributed to the lack of access to ARV for the bite victims, especially in rural areas, and the scarcity of affordable RIG in category III exposures in previously unvaccinated children. The WHO APCRI Survey 2017 also reported the problem of frequent shortages and stock outs of ERIG for PEP in India [5]. The children, in particular, are more vulnerable to severe category III bites on sensitive areas, including the head and neck region, and are more prone to deaths in the absence of the timely administration of complete PEP and RIG in category III exposures [29,34]. Therefore, the implementation of PrEP strategies over PEP can be a feasible and cost-effective solution to avert rabies deaths in school-aged children in India.

The preliminary studies on accelerated or shorter PrEP regimens involving 1-week or single day PrEP regimens have suggested their cost-effectiveness over extended regimens [36]. The WHO has also recommended the implementation of the PrEP strategy in settings with a bite incidence of more than 5%. A recent study has reported the operational feasibility of PrEP for homeless street children in India for districts having an animal bite prevalence of more than 5% [37]. However, the current study has reported the cost-effectiveness of shorter PrEP regimens (PrEP (I)) over PEP regimens at a bite incidence of 4.4% in children in the age group of 5–15 years, respectively. 

The PrEP demonstration project in school children has reported no deaths due to rabies in children in the study area in the Philippines [38]. The PrEP (I) has been reported to be cost-effective in this study. Moreover, the strategy has been advocated by various researchers in children [38,39]. Therefore, the implementation of the strategy in school-aged children can be an effective strategy in averting rabies deaths in these children. The strategy could be more cost effective if it can be implemented through the school education program, as it will reduce various direct non-medical and indirect costs, as well as operational costs.

### Limitations

There is a scarcity of nationally representative, age-specific data on animal bites and rabies in India. Therefore, the data inputs were extracted from local and regional community-based studies. There are various assumptions taken due to the dearth of literature. However, the data inputs were extracted from the closely representative studies and were validated by the field experts.

This study also did not take into consideration the regional variations and challenges in the implementation of the strategies. The operational costs of supply chain management were also not included for the estimation of costs. The cost-effectiveness analysis was conducted for the first year of implementation and the statistical cycles were not run to include the maximum protection conferred by ARV (at least 20 years). Only one bite incidence was considered for a bite victim in one year of analysis. The study is silent about the variations in health budgets, logistics, and resource procurement for rabies control in the respective states.

## 5. Conclusions

The shorter PrEP regimens are associated with a significant reduction in deaths and DALYs due to rabies in school-aged children in India. The study reported that shorter PrEP regimens are cost-effective over PEP regimens to avert deaths caused by rabies in children in India. There is a need to conduct feasibility and acceptability studies of PrEP in districts with a high prevalence of animal bites and a lower accessibility of PEP services after exposure. There is also a need to conduct these demonstration studies in rural, tribal, and hilly areas and districts under a thick cover of forests. The primary studies can be conducted in primary settings to extract data for cost-effectiveness studies of shorter regimens in real-time settings.

## Figures and Tables

**Table 1:** Vaccine Schedule for Intervention and Comparator Arms.

Arms	Strategy	Regimens	Guidelines
Intervention (PrEP I)	PrEP (ID); PEP (ID)	ARV at Day 0, 7 (2-site); ARV at Day 0 (4 site)	WHO Guidelines [11]
Comparator 1 (C1)	PrEP (ID); PEP (ID)	ARV at Day 0, 7, 21 (1-site); ARV at Day 0, 3 (1-site)	National Guidelines [12]
Comparator 2 (C2)	PrEP (IM); PEP (IM)	ARV at Day 0, 7 (1-site); ARV at Day 0, 3 (1-site)	WHO Guidelines [11]
Comparator 3 (C3)	PrEP (IM); PEP (IM)	ARV at Day 0, 7, 21 (1-site); ARV at Day 0, 3 (1-site)	National Guidelines [12]
Comparator 4 (C4)	PEP only (ID)	ARV at Day 0,3,7 (2-site) + RIG in Cat III exposure	WHO Guidelines [11]
Comparator 5 (C5)	PEP only (ID)	ARV at Day 0, 3, 7, 28 (2-site) + RIG in Cat III exposure	National Guidelines [12]
Comparator 6 (C6)	PEP only (IM)	ARV at Day 0, 3, 7, 14-28 (1-site) + RIG in Cat III exposure	WHO Guidelines [11]
Comparator 7 (C7)	PEP only (IM)	ARV at Day 0 (2-site) + Day 7, 21 (1-site) + RIG in Cat III exposure	WHO Guidelines [11]
Comparator 8 (C8)	PEP only (IM)	ARV at Day 0, 3, 7, 14, 28 (1-site) + RIG in Cat III exposure	National Guidelines [12]

ARV = anti-rabies vaccination; ID = intradermal; IM = intramuscular; INR = Indian national rupee PrEP = Pre-exposure prophylaxis; PEP = post-exposure prophylaxis; RIG = rabies immunoglobulin.

**Table 2:** Data Inputs for Model.

Data Inputs	Values		Source
Epidemiology			
Population cohort	1,000,000		Estimates
Population age group	5–15 years		Objective of the study
Annual bite incidence	4.40%		N Agarwal 2015 [13]
Probability of bite from a rabid animal	0.295		Assumptions from WHO APCRI Survey 2017(Household Survey) [5]
Prevalence of category of exposure	Cat I= 0.53% Cat II= 26.71% Cat III= 72.76%		Nandimath 2019 [14]
Probability of developing rabies in Cat I exposure from rabid animal	0.00		Expert opinion
Probability of developing rabies in Cat II/III exposure in absence of administration or full administration of PEP vaccination	0.19		Shim 2009 [15]
Probability of developing rabies in Cat III exposure in absence of administration of RIG in previously unimmunized individuals	0.30		Expert opinion
RIG administration in Cat III exposures	13.6%		WHO APCRI Survey 2017 (Community Survey) [5]
Practice of seeking PEP after exposure to animal bite	88.9 %		WHO APCRI Survey 2017 (Community Survey) [5]
Compliance for full course of ID PEP	Intervention Comparator 1 Comparator 4 Comparator 5	= 100% = 94.4% = 90.7% = 85.1%	Assumptions for regimens from WHO APCRI Survey 2017 [5]
Compliance for full course of IM PEP	Comparator 2 Comparator 3 Comparator 6 Comparator 7 Comparator 8	= 99.4% = 99.4% = 80.3% = 65.9% = 65.9%	Assumptions for regimens from WHO APCRI Survey 2017 [5]
Duration of protection	20 years		Suwansrinon 2006 [16]
Average time a person lives after the onset of rabies	5 days		Expert opinion
Disability weight (rabies)	0.655		Ock 2019 [28]
Costs & Resource Utilization			
Amount of eRIG required for children 5–15 years for local + systemic wound infilteration	4.33 mL		Calculated from Population Projections for India and States 2011–2036 [17] and Khadilkar V 2019 [19]
Amount of eRIG required for children 5–15 years for local wound infilteration only	2.64 mL		Calculated from Bharti 2016 [29]
Cost per dose of vaccine (1 mL)	INR 250		Rate Contract 2020, MP Aushadhi [20]
Cost per eRIG vial (5 mL)	INR 313		WHO APCRI Survey 2017 [5]
Cost per hRIG vial (2 mL, 300 IU)	INR 3700		WHO APCRI Survey 2017 [5]
Cost per vial of monoclonal antibodies (2.5 mL)	INR 1970		WHO APCRI Survey 2017 [5]
Hospital charges	INR 3		WHO APCRI Survey 2017 [5]
Other medicines and consumables for PEP (1st visit only)	INR 169.8		Calculated from WHO APCRI Survey 2017 [5]
Cost of syringe (per unit)	INR 2.5		Procurement costs (email communication)
Cost of gloves (per pair)	INR 14		Procurement costs (email communication)
Cost associated with human resources per patient’s visit	INR 87.39		Calculated from data from ARV clinic, ShimLa (email communication) National Health System Cost Database of India [21]
Travel cost for the patient/client and one attendant (per visit)	INR 50		WHO APCRI Survey 2017 [5]
Meal cost for the patient/client and one attendant (per visit)	INR 40		WHO APCRI Survey 2017 [5]
Loss of wages for one attendant (per visit)	INR 232		Average state-wise per day wage rate for unskilled manual workers under MGNREGA [24]
Treatment cost of fatal symptomatic rabies (per day)	INR 3600		Cost for intensive care services without ventilator as per PMJAY health benefit package 2.0 [22]
Program management cost (per capita)	INR 0.2		Calculated from Abbas 2014 [23]
Wastage Factor			
Vaccination	30%		Abbas 2014 [23]
RIG	15%		Abbas 2014 [23]
Discount Rates			
Costs	3%		WHO 2008 [26]
Utilities	3%		WHO 2008 [26]

ID = intradermal; IM = intramuscular; INR = Indian national rupee; PEP = post-exposure prophylaxis; ARV = anti-rabies vaccination; RIG = rabies immunoglobulins.

**Table 3:** Results of cost-effectiveness analysis.

	PrEP (I)	Comparator 1	Comparator 2	Comparator 3	Comparator 4	Comparator 5	Comparator 6	Comparator 7	Comparator 8
Regimen	PrEP + PEP	PrEP + PEP	PrEP + PEP	PrEP + PEP	PEP	PEP	PEP	PEP	PEP
Route of administration	ID	IM	IM	ID	ID	ID	IM	IM	IM
Discounted Costs (in INR)									
ARV (PrEP)	71,980,000	53,990,000	359,918,000	539,877,000	-	-	-	-	-
ARV (PEP)	2,800,670	1,400,335	14,004,049	14,004,049	4,201,215	5,601,729	28,008,255	28,008,255	35,010,318
RIG	-	-	-	-	754,303	754,303	754,303	754,303	754,303
Direct medical costs	207,766,892	254,274,555	509,376,347	752,765,347	15,260,138	19,128,523	41,535,049	39,120,745	51,005,110
Direct non-medical costs	113,421,127	170,471,692	114,191,833	170,475,636	6,573,676	8,763,085	8,763,085	6,573,676	10,952,494
Indirect costs	261,918,250	395,376,500	266,916,500	395,376,500	14,994,750	19,993,001	19,993,001	14,994,750	24,991,251
Treatment costs for fatal rabies	2,720,882	3,936,807	2,850,448	2,850,448	25,155,698	25,604,195	25,982,926	27,139,052	27,139,052
Health Outcomes									
Deaths (per million population)	273	395	286	286	2524	2569	2607	2723	2723
DALYs (discounted)	9479.09	13,715.17	9930.48	9930.48	87,638.22	89,200.71	90,520.14	94,547.89	94,547.89
Base Case Analysis									
Quasi-Societal Perspective									
Incremental costs (INR)		−230,303,107	−288,236,917	−716,366,906	568,045,594	567,897,297	567,591,257	567,730,161	567,367,844
Incremental costs (USD)		−12,167,324	−15,228,070	−37,846,941	30,010,862	30,003,027	29,986,858	29,994,197	29,975,055
ICER (INR/death averted)		1,887,730	22,172,071	55,105,147	−252,353	−247,342	−243,184	−231,727	−231,579
ICER (USD/death averted)		99,732	1,171,390	2,911,303	−13,332	−13,068	−12,848	−12,243	−12,235
ICER (INR/DALYs averted)		54,367.05	638,560.49	1,587,040.29	−7267.81	−7123.50	−7003.75	−6673.78	−6669.52
ICER (USD/DALYs averted)		2872.31	33,736.29	83,846.17	−383.97	−376.35	−370.02	−352.59	−352.36
		Dominant	Dominant	Dominant	Non-dominant	Non-dominant	Non-dominant	Non-dominant	Non-dominant
Cost-effectiveness		Cost effective	Not cost effective	Not cost effective	Very cost effective	Very cost effective	Very cost effective	Very cost effective	Very cost effective
Quasi-Health Systems’ Perspective									
Incremental costs (INR)		−45,467,834	−287,131,113	−531,531,639	198,792,933	198,743,514	198,437,261	198,476,552	198,312,578
Incremental costs (USD)		−2,402,147	−15,169,649	−28,081,765	10,502,585	10,499,974	10,483,794	10,485,870	10,477,207
ICER (INR/death averted)		372,687.16	22,087,008.68	40,887,049.15	−88,313.16	−86,560.76	−85,020.25	−81,010.84	−80,943.91
ICER (USD/death averted)		19,689.73	1,166,896.06	2,160,135.73	−4665.74	−4573.16	−4491.77	−4279.95	−4276.41
ICER (INR/DALYs averted)		10,733.47	636,110.69	1,177,555.97	−2543.44	−2492.97	−2448.60	−2333.13	−2331.20
ICER (USD/DALYs averted)		567.07	33,606.86	62,212.38	−134.37	−131.71	−129.36	−123.26	−123.16
		Dominant	Dominant	Dominant	Non-dominant	Non-dominant	Non-dominant	Non-dominant	Non-dominant
Cost-effectiveness		Very cost effective	Not cost effective	Not cost effective	Very cost effective	Very cost effective	Very cost effective	Very cost effective	Very cost effective

ARV = anti-rabies vaccine; DALY = disability-adjusted life years; ICER = incremental cost-effectiveness ratio; INR = Indian national rupee; PrEP = pre-exposure prophylaxis; PEP = post-exposure prophylaxis; QALY = quality-adjusted life years; USD = US dollars.

**Table 4:** Results of Scenario Analyses.

	Comparator 4	Comparator 5	Comparator 6	Comparator 7	Comparator 8
Regimen	PEP	PEP	PEP	PEP	PEP
Route of administration	ID	ID	IM	IM	IM
Scenario 1 (Local Wound Infilteration of ERIG)					
Quasi-Societal Perspective					
ICER (INR/DALYs averted)	−7267.82	−7123.51	−7003.76	−6673.78	−6669.53
ICER (USD/DALYs averted)	−383.97	−376.35	−370.02	−352.59	−352.36
Quasi-Health Systems’ Perspective					
ICER (INR/DALYs averted)	−2543.45	−2492.97	−2448.61	−2333.14	−2331.21
ICER (USD/DALYs averted)	−134.37	−131.71	−129.36	−123.26	−123.16
Scenario 2 (Use of HRIG)					
Quasi-Societal Perspective					
ICER (INR/DALYs averted)	−6634.01	−6534.25	−6448.3	−6219.27	−6215.31
ICER (USD/DALYs averted)	−350.49	−345.22	−340.67	−328.58	−328.37
Quasi-Health Systems’ Perspective					
ICER (INR/DALYs averted)	−2321.54	−2286.67	−2254.33	−2174.17	−2172.38
ICER (USD/DALYs averted)	−122.65	−120.81	−119.1	−114.87	−114.77
Scenario 3 (Use of R-Mab)					
Quasi-Societal Perspective					
ICER (INR/DALYs averted)	−6418.01	−6331.89	−6258.23	−6058.01	−6054.14
ICER (USD/DALYs averted)	−339.08	−334.53	330.63	−320.06	−319.85
Quasi-Health Systems’ Perspective					
ICER (INR/DALYs averted)	−2245.97	−2215.87	−2187.9	−2117.82	−2116.07
ICER (USD/DALYs averted)	−118.66	−117.07	−115.59	−111.89	−111.8

DALY = disability-adjusted life years; ERIG = equine rabies immunoglobulin; HRIG = human rabies immunoglobulin; ICER = incremental cost-effectiveness ratio; INR = Indian national rupee; QALY = quality-adjusted life years; R-Mab = rabies monoclonal antibodies; USD = US dollars.

**Scheme A1:**
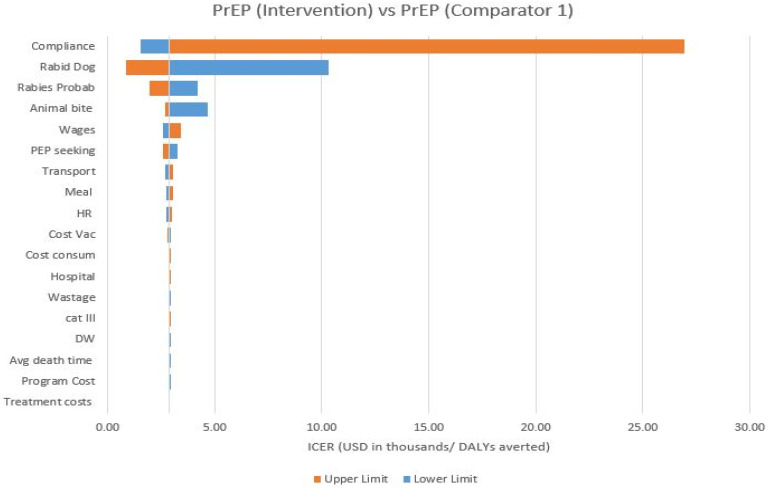
One−way sensitivity of CE analysis of PrEP (I) vs. C1.

**Scheme A2:**
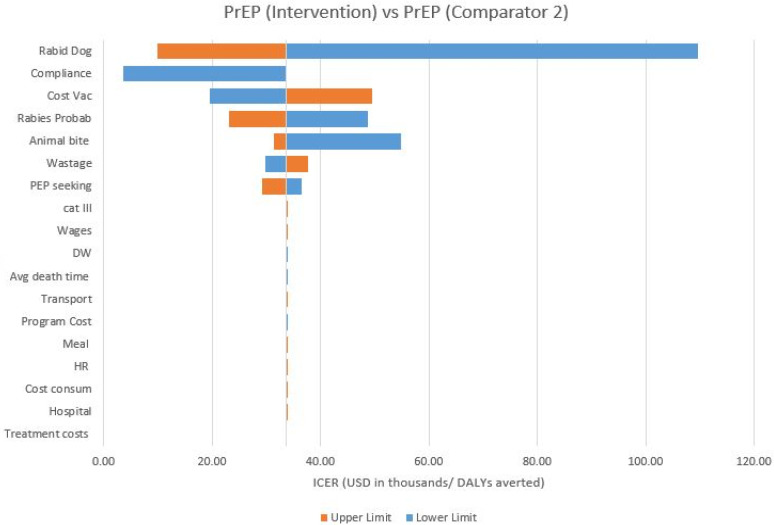
One−way sensitivity of CE analysis of PrEP (I) vs. C2.

**Scheme A3:**
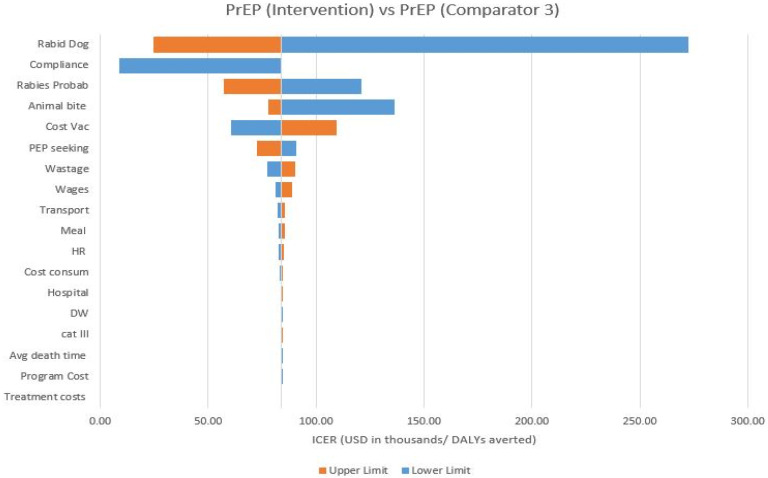
One−way sensitivity of CE analysis of PrEP (I) vs. C3.

**Scheme A4:**
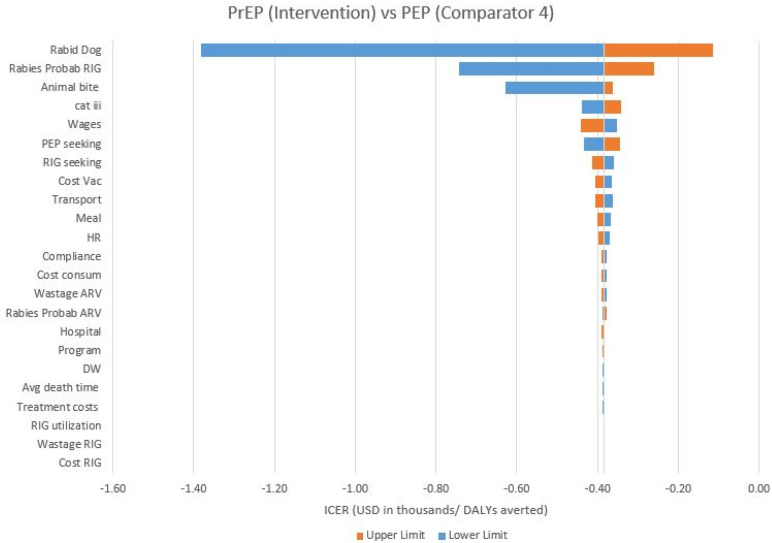
One−way sensitivity of CE analysis of PrEP(I) vs. C4.

**Scheme A5:**
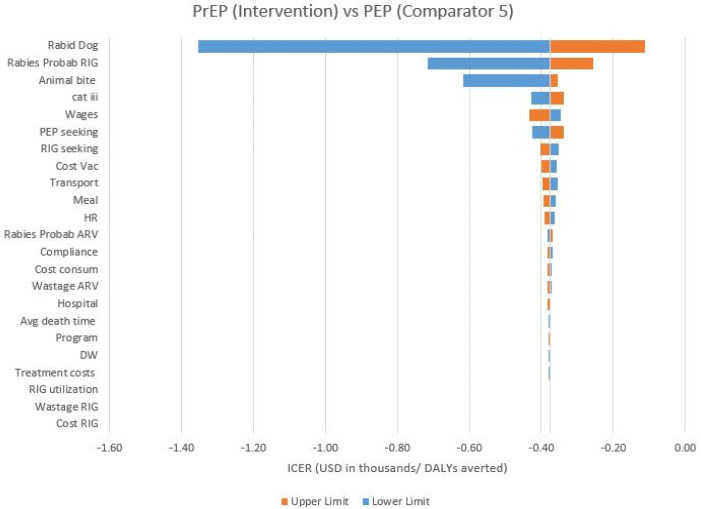
One−way sensitivity of CE analysis of PrEP (I) vs. C5.

**Scheme A6:**
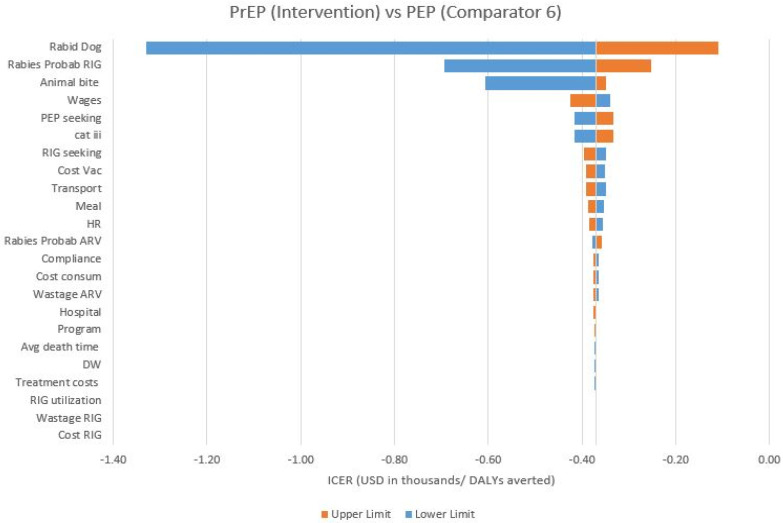
One−way sensitivity of CE analysis of PrEP (I) vs. C6.

**Scheme A7:**
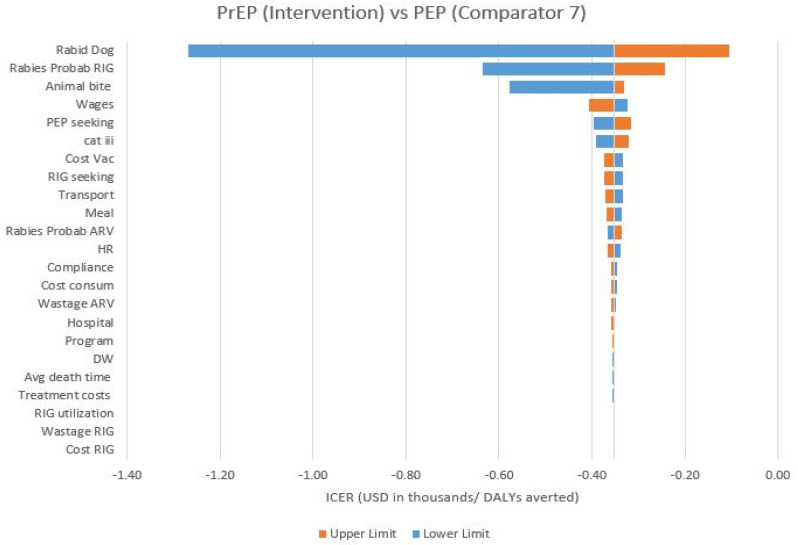
One-way sensitivity of CE analysis of PrEP (I) vs. C7.

**Scheme A8:**
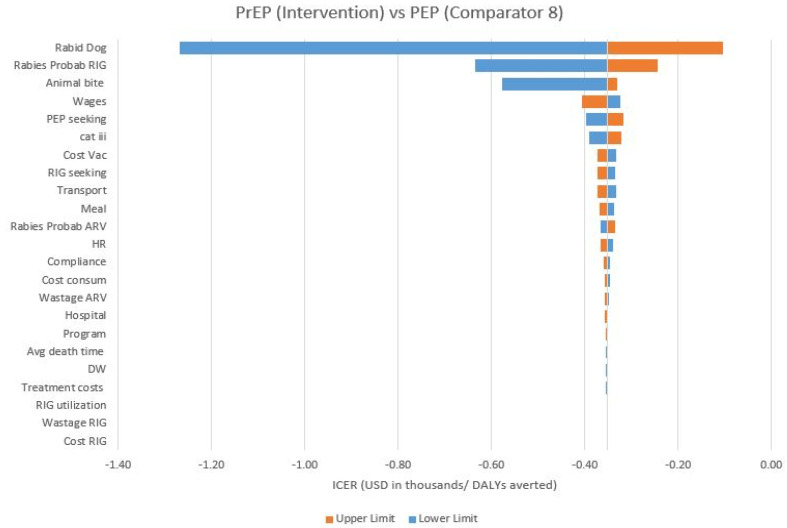
One−way sensitivity of CE analysis of PrEP (I) vs. C8. Animal bite—Animal bite incidence; Avg death time—Average time a person lives after the onset of rabies; Cat iii—Category of exposure; Compliance—Compliance for full course of PEP; Cost consum—Cost of consumables; Cost RIG—Cost per RIG vial; Cost vac—Cost per vaccine vial; DW—Disability weight; Hospital—Hospital charges; HR—Costs associated with human resources; Meal—Meal cost for the patient/client and one attendant (per visit); PEP seeking—PEP seeking behaviour of the bite victims; Program—Program management costs; Rabid dog—Rabies positivity of the biting animal; Rabies Prob ARV—Probability of developing rabies in Cat II/III exposure in absence of administration or full administration of PEP vaccination; Rabies Prob RIG—Risk probability of rabies in category III exposures in previously unvaccinated children in absence of RIG; RIG seeking—RIG administration in Cat III exposures; RIG utilization—Utilization of calculated dose of RIG; Transport—Travel cost for the patient/client and one attendant (per visit); Treatment—Treatment cost of fatal symptomatic rabies; Wages—Loss of wages of attendant; Wastage ARV—ARV wastage; Wastage RIG—RIG wastage.

## Data Availability

This study is based on a decision tree modelling adapted from the existing models used in the estimation of the global burden of rabies, and the data inputs are extracted from the existing literature or programmatic data/national surveys.

## Appendix A

**Table A1:** Data inputs for one-way sensitivity and scenario analysis (children in age group 5–15 years) Part-1.

		Value		Source	Explanations	Calculations
Population cohort		1,000,000			Estimates for decision tree	
Cohort age group		5–15 years				
Population 5–15 years (2021)		233,436,000		Population Projections for India and States 2011–2036		
Annual Bite Incidence						
Base case		4.4%		N Agarwal (2015)	(Proxy) 1 year dog-bite incidence in < 5 years age group in urban settings of Patna in a community based study	46/1045 = 4.4%
Lower limit		2.69%		N Agarwal (2004)	(Proxy) 1 year dog-bite incidence in 5–14 years age group in rural settings of Ballabgarh in a community based study	6/223 = 2.69%
Upper limit		4.68%		Vernekar (2018)	(Proxy) 1 year dog-bite incidence in 6–16 years age group in rural settings of Goa in a community based study	6–10 = 13/163; 11–16 = 3/179 => 6–16 = 16/342 = 4.68%
Probability of Bite from a Rabid Animal						
Base case		0.295		WHO APCRI Survey 2017	29.5% of the biting animals showed some signs of suspected rabies in health facility survey	
Lower limit		0.082		WHO APCRI Survey 2017	(Proxy) suspected dog rabies incidence	12/146 = 8.2%
Upper limit		1		WHO APCRI Survey 2017	All the biting animals were suspected rabid in community survey	
Probability of Developing Rabies in Cat I Exposure from Rabid Animal						
Base case		0		Expert opinion		
Probability of Developing Rabies in Cat II/III Exposure in Absence of Administration or Full Administration of PEP Vaccination						
Base case		0.19		Shim (2009)		
Lower limit		0.13		Shim (2009)	Lower value of binomial confidence intervals reported in the study	
Upper limit		0.28		Shim (2009)	Upper value of binomial confidence intervals reported in the study	
Probability of Developing Rabies in Cat III Exposure in Absence of Administration of RIG in Previously Unimmunized Individuals						
Base case		0.3		Expert opinion		
Lower limit		0.15		Calculation	(−50% of the base case value)	
Upper limit		0.45		Calculation	(+50% of the base case value)	
RIG Administration in Cat III Exposures						
Base case		13.60%		WHO APCRI Survey 2017	Community Survey	Number of Cat III victims received ERIG = 3; Number of Cat III victims received HRIG = 1; Total number of victims = 22; For ERIG = 3/22 = 13.6%; For HRIG = 1/22 = 4.5%
Lower limit		6.80%		Calculation	(−50% of the base case value)	
Upper limit		20.40%		Calculation	(+50% of the base case value)	
Practice of Seeking PEP after Exposure to Animal Bite						
Base case		88.90%		WHO APCRI Survey 2017	Community survey	
Lower limit		78.90%		Calculation	(−10% of the base case value)	
Upper limit		98.90%		Calculation	(+10% of the base case value)	
Duration of Protection						
Base case		20 years		Suwansrinon (2006)		
Average Time a Person Lives after the Onset of Rabies						
Base case		5		Expert opinion		
Lower limit		3		Calculation	(−40% of base case value)	
Upper limit		7		Calculation	(+40% of base case value)	
Disability Weight						
Base case		0.655		Ock (2019)		
Lower limit		0.133		Global burden of diseases		
Upper limit		0.797		Ock (2016)		
	Value			Source	Explanations	Calculations
Category of Bite						
	Cat I	Cat II	Cat III			
Base case	0.53%	26.71%	72.76%	Nandimath (2019)	(Proxy) clinic based study (age specific data) on dog-bitten children below 15 years	Cat I = 3/569 = 0.53%; Cat II = 152/569 = 26.71%; CatIII = 414/569 = 72.76%
Lower limit	0.53%	36.71%	62.76%	Calculations	(−10% in Cat III and subsequent +10% in Cat II)	
Upper limit	0.53%	16.71%	82.76%	Calculations	(+10% in Cat III and subsequent −10% in Cat II)	

**Table A2:** Data inputs for one-way sensitivity and scenario analysis (children in age group 5–15 years) Part-2.

	Value					Source	Explanation
Compliance for full course of ID PEP							
	Intervention	Comparator 1	Comparator 4	Comparator 5			
Base case	100.00%	94.40%	90.70%	85.10%		WHO APCRI Survey 2017	(Proxy) compliance of bite victims according to day for 4-day regimen
Lower limit	95.00%	89.40%	85.70%	80.10%		Calculation	(−) 5% of base case value
Upper limit	100.00%	99.40%	95.70%	90.10%		Calculation	(+) 5% of base case value
Compliance for full course of IM PEP							
	Comparator 2	Comparator 3	Comparator 6	Comparator 7	Comparator 8		
Base case	99.40%	99.40%	80.30%	65.90%	65.90%	WHO APCRI Survey 2017	(Proxy) compliance of bite victims according to day for 5-day regimen
Lower limit	94.40%	94.40%	75.30%	60.90%	60.90%	Calculation	(−) 5% of base case value
Upper limit	100.00%	100.00%	85.30%	70.90%	70.90%	Calculation	(+) 5% of base case value

**Table A3:** Data inputs for one-way sensitivity and scenario analysis (children in age group 5–15 years) Part 3.

	Value	Source	Explanations	Calculations
Amount of RIG required for children 5–15 years for local wound + systemic infilteration				
Base case	4.33 mL	Calculations	Population Projections for India and States 2011–2036 and Khadilkar (2019)	Age and gender specific proportionate weights and maximum dose calculations using NFHS-4 data and age specific weights in both the genders from Khadilkar V (2019)
Lower limit	2.165 mL	Calculations	(−50% of base case)	
Upper limit	6.495 mL	Calculations	(+50% of base case)	
Amount of RIG required for children 5–15 years for local wound infilteration				
Scenario 1	2.64 mL	Calculations	Bharti (2016)	For 5–10 years, Avg quantity = 2 mL No. of patients = 40, Total eRIG consumed = 2 × 40 = 80 mL For 10–15 years, Avg quantity = 3.5 mL, No. of patients = 30, Total eRIG consumed = 3.5 × 30 = 105 mL Total eRIG consumed (5–15 years) = 80 + 105 = 185 mL Average eRIG consumed = 185/70 = 2.64 mL
RIG access	13.60%	WHO APCRI Survey 2017	Assumptions from local + systemic infilteration from community survey	
Cost per dose of vaccine (per dose)				
Base case (INR 2020)	INR 250	Procurement cost	Rate Contract (2020), Madhya Pradesh Aushadhi	
Lower limit (INR 2017)	INR 128	WHO APCRI Survey 2017	Average cost of vaccine purchased in study states	
Upper limit (INR 2017)	INR 325	WHO APCRI Survey 2017	Market cost per vial: Indian Immunologicals Ltd.	
Cost per eRIG vial; 5 mL				
Base case (INR 2017)	INR 313	WHO APCRI Survey 2017	Average cost of ERIG purchased in study states	
Lower limit (INR 2020)	INR 210	Rate Contract (2020), MP Aushadhi	Procurement cost in MP	
Upper limit (INR 2017)	INR 476	WHO APCRI Survey 2017	Market cost per vial: Bharat Serums & Vaccines Ltd.	
Cost per HRIG vial (2 mL, 300 IU) (INR 2017)				
Scenario 2	INR 3700	WHO APCRI Survey 2017	Average cost of the procurement value	Brand 1 = 3749, Brand 2 = 3650; Average = 3699.5 ~ 3700
RIG access	4.50%	WHO APCRI Survey 2017	HRIG Administration in community survey	
Cost per vial of monoclonal antibodies (2.5 mL) (INR 2017)				
Scenario 3	INR 1970	WHO APCRI Survey 2017	Market price as R-Mab in market surveillance phase	
R-Mab Access	1%	Assumptions	Market Surveillance Phase	
Hospital charges (INR 2017)				
Base case	INR 3	Government Facility, WHO APCRI Survey 2017 (Median Cost)		
Lower limit	INR 2	Government Facility, WHO APCRI Survey 2017 (Q1 of IQR)		
Upper limit	INR 10	Government Facility, WHO APCRI Survey 2017 (Q3 of IQR)		
Cost of syringe (per unit) (INR 2020)				
Base case	INR 2.5	Procurement costs (2020)		
Lower limit	INR 1.25	Calculations	−50% of the base case value)	
Upper limit	INR 3.75	Calculations	(+50% of the base case value)	
Cost of gloves (per pair) (INR 2020)				
Base case	INR 14	Procurement costs (2020)		
Lower limit	INR 7	Calculations	(−50% of the base case value)	
Upper limit	INR 21	Calculations	(+50% of the base case value)	
Other medicines and consumables for PEP (1st visit only) (INR 2020)				
Base case	INR 169.8	Calculated from WHO APCRI Survey 2017		Cost reported in survey (inclusive of syringe + gloves) = 186.27; 186.27 − (2.5 + 14) = 169.77
Lower limit	INR 84.9	Calculations	(−50% of the base case value)	169.77 × 0.5 = 84.885
Upper limit	INR 254.7	Calculations	(+50% of the base case value)	169.77 × 1.5 = 254.655
Costs associated with human resources per visit (INR 2018)				
Base case	INR 87.39	Calculations	National Health System Cost Database of India	
Lower limit	INR 69.91	Calculations	(−20% of the base case value)	
Upper limit	INR 104.87	Calculations	(+20% of the base case value)	
Travel cost for the patient/client and one attendant (per visit) (INR 2017)				
Base case	INR 50	WHO APCRI Survey 2017		
Lower limit	INR 25	Calculations	(−50% of the base case value)	
Upper limit	INR 75	Calculations	(+50% of the base case value)	
Meal cost for the patient/client and one attendant (per visit) (INR 2017)				
Base case	INR 40	WHO APCRI Survey 2017		
Lower limit	INR 20	Calculations	(−50% of the base case value)	
Upper limit	INR 60	Calculations	(+50% of the base case value)	
Loss of wages for one attendant (per visit) (INR 2020)				
Base case	INR 232	Average state-wise per day wage rate for unskilled manual workers under MGNREGA		
Lower limit	INR 190	Lower limit of range reported in state-wise wage rate for unskilled manual workers (per day) under MGNREGA		
Upper limit	INR 309	Upper limit of range reported in state-wise wage rate for unskilled manual workers (per day) under MGNREGA		
Treatment cost of fatal symptomatic rabies (per day)				
Base case	INR 3600	Cost for intensive care services without ventilator as per PMJAY health benefit package 2.0		
Lower limit	INR 1800	Calculations	(−50% of the base case value)	
Upper limit	INR 5400	Calculations	(+50% of the base case value)	
Program management cost (per capita) (INR 2014)				
Base case	INR 0.2	Calculated from Abbas (2014)		Program management costs (TN) = 14,388,443.689; Total population = 72,138,958; Per capita = 14,388,443.689/72,138,958 = 0.2
Lower limit	INR 0.1	Calculations	(−50% of the base case value)	
Upper limit	INR 0.3	Calculations	(+50% of the base case value)	
Wastage factor vaccine				
Base case	30%	Abbas (2014)		
Lower limit	15%	Calculations	(−50% of the base case value)	
Upper limit	45%	Calculations	(+50% of the base case value)	
Wastage factor RIG				
Base case	15%	Abbas (2014)		
Lower limit	7.5%	Calculations	(−50% of the base case value)	
Upper limit	22.5%	Calculations	(+50% of the base case value)	
Discount rates				
Cost	3%	WHO (2008)		
Utility	3%	WHO (2008)		

Calculations for ERIG (Children in Age Group 5–15 Years)

**Table A4:** Age specific weighted weight.

Age (yrs)	Projected Total Number of Children 2021 (‘000) [17]	Projected Number of Boys 2021 (‘000) [17]	50th Percentile Weight in Kgs in Boys in India (LMS Method) [19]	(Weighted) Proportionate Weight (Boys)—PWB in Kgs	Projected Number of Girls 2021 (‘000) [17]	50th Percentile Weight in Kgs in Girls in India (LMS Method) [19]	(Weighted) Proportionate Weight (Girls)—PWG in Kgs	Age Specific Weighted Weight—AW in Kgs
	T	B	WB	PWB= WB × (B/T)	G	WG	PWG = WG × (G/T)	AW = PWB + PWG
5	22,909	12,122	17	8.995329347	10,787	16.5	7.769239164	16.8
6	22,957	12,175	19.3	10.23554907	10,782	18.5	8.688722394	18.9
7	23,033	12,210	21.7	11.50336474	10,823	20.9	9.820722442	21.3
8	23,137	12,227	24.6	13.00013831	10,910	23.9	11.26978433	24.3
9	23,268	12,225	27.8	14.6061114	11,043	26.9	12.76674832	27.4
10	23,346	12,171	30.9	16.10913647	11,175	30.7	14.69512979	30.8
11	23,406	12,129	34.3	17.77427583	11,277	35	16.86298385	34.6
12	23,546	12,166	38.6	19.9442623	11,380	39.5	19.09071605	39.0
13	23,766	12,282	43.2	22.3252714	11,484	43.4	20.97137087	43.3
14	24,067	12,477	48.4	25.09190177	11,590	46.5	22.3931109	47.5
15	24,419	12,699	53.1	27.61443548	11,720	48.5	23.2777755	50.9

Total number of children 5–15 years in ‘000 (T’) = 257854.

**Table A5:** Combined Age specific weighted weight.

Age	Number of Children	Age Specific Proportion	Weighted Age Specific Weight	
	C	A = C/T’	AW	A × AW
5	22,909	0.08884485	16.8	1.49
6	22,957	0.089031002	18.9	1.68
7	23,033	0.089325742	21.3	1.90
8	23,137	0.089729071	24.3	2.18
9	23,268	0.090237111	27.4	2.47
10	23,346	0.090539608	30.8	2.79
11	23,406	0.090772298	34.6	3.14
12	23,546	0.09131524	39.0	3.56
13	23,766	0.092168436	43.3	3.99
14	24,067	0.093335764	47.5	4.43
15	24,419	0.094700877	50.9	4.82
Proportionate (weighted) weights in 5–15 years (kgs)				32.47

**Table A6:** Calculations for ERIG, HRIG & R-Mab for Scenario Analysis.

Amount Calculations for RIG (Scenario Analysis)				
	ERIG (Base Case)	ERIGLocal Only (Scenario 1)	HRIG (Scenario 2)	R-Mab (Scenario 3)
Vial	5 mL	5 mL	2 mL	2.5 mL
Discounted cost per vial (INR)	195.66	195.66	2312.84	1231.43
Potency (per mL)	300 IU/mL	300 IU/mL	150 IU/mL	40 IU/mL
Potency (per vial)	1500 IU/vial	1500 IU/vial	300 IU/vial	100 IU/vial
Dose	40 IU/kg wt	40 IU/kg wt	20 IU/kg wt	3.33 IU/kg wt
	1 mL = 300 IU	1 mL = 300 IU	1 mL = 150 IU	1 mL = 40 IU
Maximum dose calculation	Dose = 40 U × 32.47= 1298.8 IU		Dose = 20 IU × 32.47= 649.4 IU	Dose = 3.33 IU × 32.47 = 108.13 IU
(Weighted proportionate weight for cohort)	Amount = 1298.8/300 mL		Amount = 649.4/150 mL	Amount = 39.1/40 mL
	Amount = 4.33 mL	Amount = 2.64 mL [33]	Amount = 4.33 mL	Amount = 2.7 mL
Amount of RIG required in children	4.33 mL	2.64 mL	4.33 mL	2.7 mL
Multiplication factor for use from a vial	4.33/5 = 0.866	2.64/5 = 0.528	4.33/2 = 2.165	2.7/2.5 = 1.08

**Table A7:** Sensitivity Analysis for RIG.

Amount of RIG for 5–15 Years of Children (One-Way Sensitivity: Local + Systemic Infiltration)			
	Amount	Multiplication Factor	Calculations
Base case	4.33 mL	4.33/5 = 0.866	
Lower limit	2.165 mL	2.165/5 = 0.433	(−50% of base case)
Upper limit	6.495 mL	6.495/5 = 1.299	(+50% of base case)

Cost Associated with Human Resource

**Table A8:** Total number of visits.

Anti Rabies Vaccination (ARV) Clinic, Shimla *			
Year	Number of New Patients	Visits/Patient	Total Visits
2015	2724	4	10,896
2016	2789	4	11,156
2017	2794	4	11,176
	Total number of visits in ARV clinic		33,228
	Average per year visit in ARV clinic (V)		11,076

* Data from Anti-Rabies Clinic Shimla through email communication

**Table A9:** Cost per patient visit in ARV clinic.

District Hospital, Himachal Pradesh				
	Formula	Doctor	Staff Nurse	Support Staff
Average per month salary (INR 2018) [21]	S	96,085	38,663	22,780
Average per year salary (INR 2018)	S × 12	1,153,020	463,956	273,360
Time contributed to ARV clinic	*t*	20%	100%	100%
Proportionate per year salary (INR 2018)	P = (S × 12) × *t*	230,604	463,956	273,360
Total number of patient visits/year	V	11,076	11,076	11,076
Cost per patient visit in ARV clinic (INR 2018)	P/V	20.82	41.89	24.68

Human resource cost incurred per visit (INR 2018) = 20.82 + 41.89 + 24.68 = INR 87.39.

DALY Calculations

Formulae:

YLL = Number of deaths × standard life expectancy at age of death (1)

YLD = Number of new cases of a disease × disability weight × the average time a person lives with the disease before death (2)

**Table A10:** DALY Calculations for 5–15 years

	PrEP (I)	Comparator 1	Comparator 2	Comparator 3	Comparator 4	Comparator 5	Comparator 6	Comparator 7	Comparator 8
Number of deaths	273	395	286	286	2524	2569	2607	2723	2723
Life expectancy ^@^	62.7	62.7	62.7	62.7	62.7	62.7	62.7	62.7	62.7
YLL	17,117.1	24,766.5	17,932.2	17,932.2	158,254.8	161,076.3	163,458.9	170,732.1	170,732.1
Number of new cases ^%^	273	395	286	286	2524	2569	2607	2723	2723
DW	0.655	0.655	0.655	0.655	0.655	0.655	0.655	0.655	0.655
Average time before death (years) ^^^	0.0137	0.0137	0.0137	0.0137	0.0137	0.0137	0.0137	0.0137	0.0137
YLD (due to rabies)	2.4495	3.5442	2.5662	2.5662	22.6468	23.0506	23.3916	24.4324	24.4324
DALYs (undiscounted)	17,119.5495	24,770.0442	17,934.7662	17,934.7662	158,277.4468	161,099.3506	163,482.2916	170,756.5324	170,756.5324
DALYs (discounted)	9479.09	13,715.17	9930.48	9930.48	87,638.22	89,200.71	90,520.14	94,547.89	94,547.89

^@^ Standard life expectancy at the age of 10 years in India = 62.7 years [SRS based abridged life tables (2014–18), Census of India 2011. Assumption: for the cohort of 5–15 years, life expectancy is taken at the age of 10 years only. ^%^ Number of new cases of rabies = number of rabies deaths (assumption: 100% mortality in rabies). ^^^ Average time a person lives with the disease before death = 5 da ys (5/365 year).

Tornado Diagrams of CE Analysis
